# Analysis of Codon Usage Patterns in Toxic Dinoflagellate *Alexandrium tamarense* through Expressed Sequence Tag Data

**DOI:** 10.1155/2010/138538

**Published:** 2010-11-01

**Authors:** Yi-Yuong Hsiao, Chorng-Horng Lin, Jong-Kang Liu, Tit-Yee Wong, Jimmy Kuo

**Affiliations:** ^1^Graduate Institute of Marine Biotechnology, National Dong Hwa University, Pingtung 944, Taiwan; ^2^Department of Planning and Research, National Museum of Marine Biology and Aquarium, Pingtung 944, Taiwan; ^3^Department of Bioresources, Da-Yeh University, Da Tsuen, Chang hua 515, Taiwan; ^4^Department of Biological Sciences, National Sun Yat-Sen University, Kaohsiung 80424, Taiwan; ^5^Bioinformatics Program, Department of Biology, The University of Memphis, Memphis, TN 38152, USA

## Abstract

We have analyzed synonymous codon usage in the genome of *A. tamarense* CCMP 1598 for protein-coding sequences from 10865 expressed sequence tags (ESTs). We reconstructed a total of 4284 unigenes, including 74 ribosomal protein and 40 plastid-related genes, from ESTs using FrameDP, an open reading frame (ORF) prediction program. Correspondence analysis of *A. tamarense* genes based on codon usage showed that the GC content at the third base of synonymous codons (GC3s) was strongly correlated with the first axis (*r* = 0.93 with *P* < .001). On the other hand, the second axis discriminated between presumed highly and low expressed genes, with expression levels being confirmed by the analysis of EST frequencies (*r* = −0.89 with *P* < .001). Our results suggest that mutational bias is the major factor in shaping codon usage in *A. tamarense* genome, but other factors, namely, translational selection, hydropathy, and aromaticity, also appear to influence the selection of codon usage in this species.

## 1. Introduction 

Most amino acids (except Met and Trp) can be coded by more than one codon. However, codon usage by individual organisms is not random. Bias in using a particular set of codons for protein synthesis is driven by multiple factors [1]. Among these factors, mutational bias and natural selection are likely to play an important role in shaping the codon usage profile of different genes and different organisms [2]. Mutational bias can influence the whole genome and drives the change in genomic GC composition. Examples of mutational bias affecting codon usage can be illustrated in many prokaryotes with extremely GC-poor or GC-rich genome [2, 3] and in humans [4]. On the other hand, codon usage may be an important tool in regulating the efficiency and accuracy of protein synthesis. Highly expressed genes often exhibit a high degree of codon bias whereas lowly expressed genes often contain many alternative synonymous codons [5].

Dinoflagellates, a large and diverse group of eukaryotic flagellated microalgae, are important primary producers. They play an important role in the aquatic food chain in marine and fresh water environments [6]. These organisms can be living as free-living, parasitic, or endosymbiotic; phototrophic or heterotrophic; marine or freshwater individuals [7]. Several species of these eukaryotic algae, such as *Alexandrium* spp., can produce toxins and impose a huge impact on marine ecosystems [8]. Molecular evolutionary analysis suggests that dinoflagellates, together with the ciliates and apicomplexans, form a monophyletic group [9]. These organisms also display certain odd biological characteristics, such as liquid crystalline DNA in chromosome, extracellular spindle through the nuclear envelope, and permanently condensed chromosome during cell proliferation [10]. Several photosynthetic species contain peridinin, a light harvesting pigment, in their photosynthetic organelles (plastids). Most of plastid genes of peridinin-containing dinoflagellate have been discovered to transfer to the cell nucleus [11, 12], and only a small part of genes encoded in the plastid are formed as minicircles [13]. Analysis of these plastid-related genes is important since dinoflagellates are the only eukaryotes to have them in the nucleus [14].

Dinoflagellates typically possess large genomes, ranging approximately from 3 pg (similar to the size of haploid human genome) to more than 250 pg [15]. This large genomic size makes dinoflagellates unlikely to be selected for complete genome sequencing. One possible alternative to exploit the genomic organization of this organism is to analyze the already published expressed sequence tags [16]. ESTs are generated by single-pass sequencing of random cDNA clones [17]. Recently, several dinoflagellate EST libraries have been generated for various purposes [7, 11, 18, 19]. These ESTs results become a useful resource for future investigations of the coding genes at the whole-genome level.

A haploid *A. tamarense* cell contains approximately 143 chromosomes with a genome size of about 200 pg/cell. This peridinin-containing dinoflagellate is often used as a model system to help us understand toxic blooms and paralytic shellfish poisoning [18]. It is known that analysis of codon usage pattern can help us understand not only the basics of molecular biology but also the factors shaping codon usage. Although the use of bioinformatics approach to study codon usage pattern has been performed for many organisms, much less studies have been performed using EST sequence data [16, 20–22]. In this study, we investigated the codon usage profile from EST library for this interesting dinoflagellate using method of multivariate statistical analysis, with special reference to the plastid-related genes and ribosomal protein genes.

## 2. Materials and Methods

### 2.1. EST Data and Clustering

Ten thousand eight hundred and sixty-five (10,865) ESTs from the toxic dinoflagellate *A. tamarense* CCMP 1598 were retrieved from the NCBI dbEST database. These ESTs were pooled from two different libraries (nonnormalized and normalized cDNA libraries) created by the same authors [18]. Most EST data (10,770 ESTs, 98.9%) are single sequence read from the 3′ end including 3′ untranslated region (UTR). The accession numbers of these EST sequences are CF751845-CF751962, CF774560-CF774855, CF947047-CF948546, CK431405-CK433904, CK782344-CK786698, CV553867-CV555405, and CX769195-CX769771. Sequence data were clustered to obtain putatively unique transcripts (unigenes) by using the Uicluster v.2-1.1 program [23] with default parameter setting. A minimum match percentage of 95% for 40 overlapping bases was used to determine two sequences as one cluster.

### 2.2. Prediction of Coding Sequences in ESTs

The clustered EST sequences of *A. tamarense* transcripts were then analyzed by using a recently developed program FrameDP v.1.0.3 [24], a self-training integrative pipeline for predicting the position of the translated region in EST. FrameDP is based on FrameD [25] which can identify ORFs by using extended interpolated Markov models (IMMs) and has frameshift correction ability. However, unlike FrameD, FrameDP can automatically use BLASTX results to generate training sequences and then to calculate training matrix, expected to represent the coding style of the species, without human curation. A collection of putative protein-coding sequences (CDSs) of *A. tamarense* was generated based on similarity with known proteins and on coding style recognition. To improve the quality of sequences and avoid possibly false positive, only CDSs with length larger than 150 bp were used for this study. In addition, clusters predicted with more than one ORF were rejected.

### 2.3. Ribosomal and Plastid-Related Genes Identification

To identify ribosomal genes, the gene sequences were compared with the NCBI nonredundant (NR) database by using the BLASTX algorithms [26]. Queries were performed with the NCBI stand-alone BLAST program [26]. The NR database and BLAST program were downloaded in October, 2009 from NCBI. Putative ribosomal genes (*E* < 10^−5^) were selected and then confirmed manually. Putative plastid-related genes of *A. tamarense* CCMP 1598 were identified from the published literature [12].

### 2.4. Codon Usage Analysis 

The frequency of 59 codons code for 18 amino acids (excluding Met, Trp, and stop codon) was examined by using three different codon indices: relative synonymous codon usage (RSCU), GC content at the third position of synonymous codons GC3s, and effective number of codons Nc [1, 27]. RSCU is the observed frequency of a codon divided by the frequency expected if all synonyms coding for that amino acid are used equally; therefore, RSCU values close to 1.0 indicate a lack of bias for that codon. GC3s is defined as the frequency of G or C nucleotides present at the third position of synonymously variable sense codons. Nc value is used to measure the magnitude of codon bias for an individual CDS. An Nc can have a value from 20, in the case of extreme bias where each amino acid is restricted in using one particular codon, to 61 when the use of alternative synonymous codon is equally likely [27].

### 2.5. Analysis Tools

GC3s, Nc, and RSCU values for ribosomal protein genes, plastid-related genes, and other protein coding genes were calculated using the program CodonW 1.4.2 [28]. Correspondence analysis (COA) [29, 30] was performed using CodonW to demonstrate the major factor in causing codon usage variation among genes. One-way ANOVA, Pearson's correlation analysis, and *χ* ^2^ test were performed with software SPSS version 12.0.

## 3. Results

### 3.1. *Alexandrium tamarense* EST Data and Clustering

A total of 10865 *A. tamarense* ESTs that are the subject of this research were downloaded from the GenBank. The average sequence length of these ESTs, after trimming the poly(A) sequence, was 558 bp. Most of the ESTs (88.9%, 9678 ESTs) were longer than 400 bp. The global GC content for these ESTs was 58.4%. This value is similar to the results (56.4%) for dinoflagellate *Alexandrium catenella* ESTs obtained by Uribe et al. [7]. The initial ESTs were grouped into 6527 unigenes, in which 2031 of them had two or more than two ESTs whereas the remaining 4496 were singletons. BLASTX result of these 6527 unigenes shows that 2385 (36.5%) of them shared significant similarity to a protein in database with a cutoff of *E*-value <  1 × 10^−5^. The remaining 4142 (63.5%) unigenes had low similarity scores. They may relate to some novel proteins or to noncoding sequences.

### 3.2. Reconstruction of Coding Sequences in Unigenes

To analyze the codon usage patterns of *A. tamarense* genome, the coding frame and putative coding sequence on the *A*.* tamarense* unigenes were determined with FrameDP program [24]. Among the 6527 unigenes, FrameDP predicted 72.5% (4735 unigenes) having at least one CDS. The remaining unigenes may correspond to short or low-quality transcripts covered only by a single EST coverage. Only 34.9% (1652 unigenes) FrameDP predicted unigenes have a hit with the UniProt database [31] with a cutoff of *E*-value <  1 × 10^−4^. This suggests that FrameDP could efficiently extract CDSs from ESTs.

**Figure 1:**
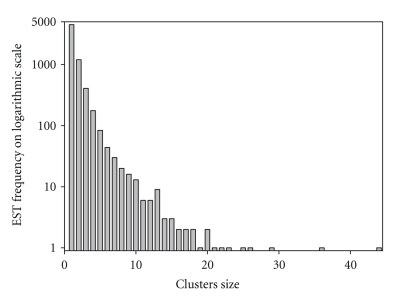
Distribution of *Alexandrium tamarense* CCMP 1598 reconstructed genes by their original cluster size.

To further increase the quality of the sequences, unigenes identified as containing more than one CDS or containing CDS with length < 150 bp were excluded from the analysis. We also excluded mitochondrial genes (6 CDSs) from the analysis, since we only consider the codon usage of nuclear protein CDSs. A total of 4284 reconstructed genes met the above criteria and were selected for further studies. The distributions of the cluster size and frequency of these reconstructed genes were shown in Figure 1. It should be noted that some of the sequences are partial even though they are referred herein as “genes”.

### 3.3. Reconstruction of Ribosomal and Plastid-Related Genes

Ribosomal protein genes of *A. tamarense* were identified by comparing the above reconstructed genes with the NR database. The products of these genes are considered as essential genes, and they often have been used to represent the highly expressed genes by most researchers in the field [16, 32]. A total of 74 ribosomal protein genes was identified in our dataset.

Hackett et al. [12] recognized 48 *A. tamarense* proteins that are of plastid function. Using the FrameDP program, more than 80% of these genes (40) were successfully reconstructed in our dataset. This result further supported that FrameDP can efficiently extract CDSs from ESTs. In general, plant and algal plastids contain a circular genome that, although varying in complexity and genetic content, is about 150 kb in size and encodes between 130–150 genes [18]. Currently, only about 16 proteins encoded on minicircles have been found in the peridinin plastid. The remaining genes required for photosynthesis are massively transferred from the plastid to the nucleus. Therefore, our reconstructed plastid-related genes might be equal to about 30%–35% of plastid genes that transfer to the nucleus.

### 3.4. Codon Usage Patterns

We divided our reconstructed genes into three groups: ribosomal protein genes, plastid-related genes, and other protein coding genes.  Table 1 showed the mean values and standard deviations of the percentage of global GC, of the percentage of GC at the first (GC1), second (GC2), and third (GC3) codon positions as well as of the effective number of codon Nc for three gene groups. The mean value of GC content for the reconstructed genes was 61.94%. This value is slightly higher than the results (60.8%) of previous research [18]. One-way ANOVA test comparing GC content between different codon positions showed that the GC content in GC3 was significantly greater (*P* < .001) than the GC contents of GC1 and GC2 for all these gene groups. The 3rd base of a codon is said to wobble, meaning that, very often, changes in the 3rd base of a codon would not change the amino acid encoded, and this reflects that base compositional mutational bias led to different codon choice within the same protein sequence [5]. However, the 1st and 2nd bases of a codon are not wobble and are subject to function constrain. Mutation in these positions will change the amino acid, and thus the function, of a protein. The significantly lower GC content at the 2nd base of a codon in *A. tamarense* is similar to that in *Drosophila melanogaster *[33], *Oryza sativa*, and *Zea mays* [34].

When different gene groups were considered, ribosomal protein genes have significantly higher (*P* < .05) GC3 values and significantly lower (*P* < .05) GC1, GC2, and Nc values than other protein-coding genes. However, all these parameters of the plastid-related genes failed to reveal statistically reliable difference with other protein coding gene groups at a significance level of 5%. The low Nc value in ribosomal protein genes suggested that highly expressed genes exhibit strong codon usage bias in *A. tamarense*. This strong codon usage bias might be the contribution of translational selection [32].

The overall codon usage of *A. tamarense* was presented in Table 2. Since this species has a moderately high GC content, it is expected that C or G ending codons would predominate. Of all the 18 degenerately encoded amino acids in Table 2, all preferentially used degenerate codons were found to be C or G ending codons. This supports the argument of mutational bias presented in Table 1. However, some variations were noticed in the RSCU values. For example, among Gly codons GGC, which ends with a C, is about 3.1 times as frequent as GGG, which ends with a G (in Table 2). This suggests that simple mutational bias cannot explain the usage of all codons.

**Figure 2:**
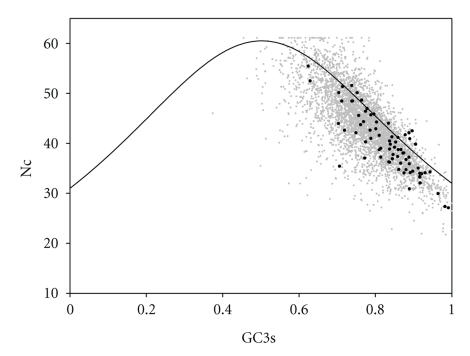
Nc-plot (Nc versus GC3s) of *Alexandrium tamarense* CCMP 1598 reconstructed genes. The solid curve represents the expected curve between GC3s and Nc under random codon usage. Gray dots and black circles indicate all genes and ribosomal protein genes, respectively.

To further analyze the degree of heterogeneity in codon usage in *A. tamarense* genomes, the GC3s and Nc values (Nc plot, a plot of Nc versus GC3s) for all the genes were calculated to determine whether codon heterogeneity exists among genes of *A. tamarense* (Figure 2). Nc plot has been used effectively by many researchers to explore the codon usage variation among genes for many different species [27]. The solid curve in Nc plot represents the expected values of Nc under totally random codon usage. If synonymous codon bias is only subject to compositional constraints, it should fall on or just below the expected curve in Nc plot. However, if synonymous codon bias is subject to natural selection, it should fall considerably below the expected curve. 

The Nc vs GC3 plot of all genes (gray dots) was shown in Figure 2. Also shown were the Nc vs GC3 plots of the ribosomal protein genes (close circles). In Figure 2, most of the genes were below the expected values except for a few genes. This indicated that this organism exhibits high variation of codon bias. In turn, it suggests that codon usage in most *A. tamarense* genes is affected not only by compositional constraints but also by other factors. Most of the ribosomal protein genes of *A. tamarense* genome clustered at the low ends (Figure 2), and only a few ribosomal protein genes were lying on or above the expected curve. Ribosomal protein genes are considered as highly expressed genes. They often exhibited significantly strong codon usage bias. This codon usage bias provides further evidence for translational selection.

### 3.5. Correspondence Analysis on Codon Usage

COA of codon usage was used to determine the major source of variation among the *A. tamarense* genes. Each gene is codified by a vector of 59 variables, which represents the number of codons for which there are synonyms. COA shows these genes in a multidimensional space of 59 axes. Among these vectors, the axes that represent the most prominent factors contributing to the variation among genes are plotted [35]. This statistical approach has been extensively used to characterize the major trends in codon usage among the genes in several species [32, 35, 36].

**Figure 3:**
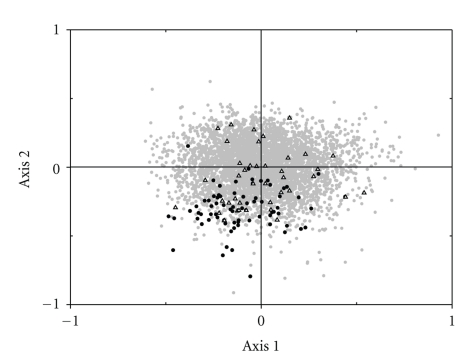
Distribution of *Alexandrium tamarense *CCMP 1598 reconstructed genes on the plane corresponds to the coordinates on the first and second axes produced by the correspondence analysis on codon usage. Gray dots, black circles, and hollow triangles indicate all genes, ribosomal protein genes, and plastid-related genes, respectively.

Figure 3 shows the plot of CDSs on the first and second major axes produced by COA on the simple codon count for *A. tamarense* genome. Also shown were the ribosomal protein genes and plastid-related genes. The variation on the first and the second dimensions explained 7.82% and 5.56% of the total codon variation, respectively. In *A. tamarense* genomes, the majority of genes near the origins of the axes clustered together to form an ellipse-shaped cloud in a range of −0.6 to +0.8 for the first axis and −0.5 to +0.5 for the second axis. The position of each gene along the first axis was strongly correlated with its GC3s value (*r* = 0.93 with *P* < .001) and Nc value (*r* = −0.75 with *P* < .001). The correlation coefficient of Nc with first axis was less than that of GC3s, and this may be due to the negative correlation between GC3s and Nc (*r* = −0.75 with *P* < .001). Our result suggested that the position of each sequence along the first axis is strongly correlated with its GC3s content. Therefore, mutational bias is the major factor in shaping codon usage in the *A. tamarense* genome.

**Figure 4:**
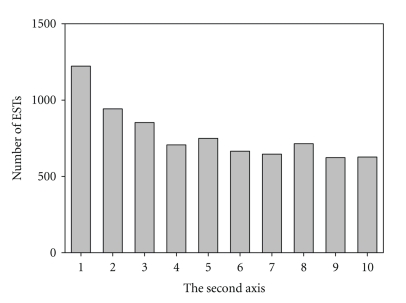
Histogram of the distribution of the number of ESTs along the second axis for *Alexandrium tamarense *CCMP 1598 reconstructed genes. The second axis was divided into 10 parts, each of them containing an equal number of genes (i.e., the genes in group 1 have the lowest values of second axis).

On the other hand, many putatively highly expressed genes, such as ATP synthases, elongation factors, heat shock proteins, and histones, were clustered on the lower part of the second axis. In addition, almost all of the ribosomal protein genes were also found to situate on the lower part of second axis, which indicated that codon bias in the ribosomal protein genes is the result of selection for translational efficiency. These results suggested that gene expression levels in *A. tamarense* genome may be responsible for the synonymous codon usage. To confirm this assumption, we counted the number of ESTs for each gene and its distribution along the second axis (Figure 4). Our result showed that the second axis in this species was negatively correlated with gene expression levels (*r* = −0.89 with *P* < .001). Therefore, this analysis clearly demonstrates that the genes with most negative values along the second axis are more highly expressed, which indicates that several codons are statistically more frequent among the highly expressed genes, and this is similar to the results observed for several cold-blooded vertebrates, such as three species of cyprinidae fish [37] as well as *Xenopus laevis* [30]. It should be noted that COA was based on codon usage rather than RSCU (Figure 3), because the second axis was more significantly correlated to gene expression levels using codon counts, although both codon usage and RSCU were significantly correlated to the second axes.

In addition, using Pearson's correlation analysis the second axis (Figure 3) was also found to correlate with hydropathy (*r* = 0.292, *P* < .001) and aromaticity (*r* = −0.268, *P* < .001) for each gene. Correlation analysis can indicate to what extent hydropathy and aromaticity are associated with the second axis. The correlation coefficient of hydropathy and aromaticity was much less than gene expression levels, suggesting that hydropathy and aromaticity play a minor role in shaping codon usage in this genome. 

Contrary to ribosomal protein genes, in Figure 3 the distribution of plastid-related genes was more dispersed. Since the second axis in *A. tamarense* is negatively correlated with gene expression, the plastid-related genes with the second axis values less than − 0.25 (10% of total genes with most negative value along the second axis) were classified as the predicted highly expressed genes. We identified 11 plastid-related genes might be putatively highly expressed genes, including heat shock protein 70, chloroplast photosystem I protein E, elongation factor Tu, chloroplast ferredoxin-NADP^+^ reductase, photosystem I assembly protein, 50S ribosomal protein L12, 40S ribosomal protein S15, oxygen evolving enhancer 1 precursor, chloroplast photosystem I subunit III, chloroplast cytochrome *f*, and photosystem I ferredoxin-binding protein.

### 3.6. Translational Optimal Codons

To understand which codons are preferred among the highly expressed sequences, we compared the codon usage patterns for 10% (428 genes each) of total genes displaying the extreme values at both ends of the second axis, and the differences between these two groups were tested with *χ* ^*2*^ test. For each codon, the *χ* ^*2*^ test can be calculated using a 2 × 2 table, in which the first row contains the values for the codon being analyzed and the second row is the total numbers of synonymous alternatives [32]. The result of this analysis was shown in Table 3. Of all the 18 amino acids, we identified 17 codons whose codon usage was significantly (*P* < .05) incremented among the highly expressed genes. According to the definition of Stenico et al. [38], these 17 codons are the translational optimal codons of this organism. They tend to have U (47.1%) and C (23.5%) in the third codon position, which is consistent with the correlation between U3 (*r* = −0.17 with *P* < .001) and C3 (*r* = −0.13 with *P* < .001) with the second axis. However, it appears that neither mutational bias (high GC3) nor translational selection (high UC3) could explain the usage of this subset of triplets. An inspection of Table 3 permits the detection of several tendencies and rules. (1) In highly expressed genes, all preferentially used codons for each amino acid were C-ending codons except GUG (Val); (2) the G-ending codons are never preferred in highly expressed genes except those belonging to sextets; (3) the NAN codons are never preferred in highly expressed genes except GAU (Asp).

## 4. Discussion

In this study, the EST-based analysis has been successful applied to elucidate the synonymous codon usage bias of dinoflagellate. A total 4284 unigenes were reconstructed from 10865 *A. tamarense* EST sequences using ORF prediction program. Reconstructing CDSs from EST data can avoid bias during analysis of codon usage better than just using sequence similarity to identified CDSs which only identified sequences with BLASTX hit [16]. Also, this method can increase the number of identified unigenes in *A. tamarense* ESTs by about 150% compared to just using sequence similarity to identified CDSs. The exact number of genes in *A. tamarense* is still unknown. According to the result of Hou and Lin [39], the *A. tamarense* genome is estimated to contain 75,000–85,000 nuclear genes based on genome size, corresponding to 1.8% and 0.05% gene-coding percentages. However, many dinoflagellate genes are highly redundant (30–5000 copies) suggesting that genome duplication is very possible [10, 40]. Our reconstruction of 4284 unigenes should be reasonably large enough to represent the overall genomic profile of this organism. However, since our analysis is based only on EST dataset, it is possible that a few lowly expressed genes were not included in the dataset that could bias our results slightly.

The GC content is one the most important features of genome. The genomic DNA of different organisms has a particular mean GC content. Previous studies have demonstrated that genes in organelles, like mitochondrion or plastid, usually have GC content much lower than genes in the nuclear genome [12, 34]. Therefore, one may expect the plastid-related genes will have lower GC content and highly bias codons comparing with protein coding genes in *A. tamarense*. However, both high mean Nc value (Table 1) and dispersed distribution for plastid-related genes (Figure 3) suggested that neither GC content nor codon usage displays significant difference between protein coding genes and plastid-related genes. Therefore, our results might suggest that *A. tamarense* have an efficient mechanism to select codons of transferred genes, like plastid-related genes, to adapt GC content and codon usage of their nuclear genome.

In contrast, the mean Nc value of ribosomal protein genes was significantly lower than the mean Nc value of protein coding genes (Table 1). In addition, most of the ribosomal protein genes of *A. tamarense* genome clustered at the low ends (Figure 3) suggested that ribosomal protein genes are considered as highly expressed genes and exhibit strong codon usage bias. This strong codon usage bias is the contribution of translational selection.

Previous studies suggested that dinoflagellates seem to have an active gene transfer mechanism in order to harbor plastids derived from different organisms [10]. Several hypotheses have been suggested why genes should be lost from plastid to nucleus [41]. One possible explanation is that genes in the plastid are exposed to high level of oxygen free radical during photosynthesis. Moving the genes to nucleus might protect plastid genes from the occurrence of deleterious mutation. Another explanation is that genes in plastid genome usually are AT-rich and subject to unfavorable mutation [42]. Moving the genes to the nucleus will avoid this tendency. If this hypothesis is true, then it is not surprising that *A. tamarense* has an efficient mechanism to select GC-rich codons for transferred genes to adapt its nuclear genome.

Synonymous codon usage bias in genes is an important evolutionary phenomenon and has been increasingly documented in a wide range of organisms from prokaryotes to eukaryotes. Apart from natural selection and mutational bias, many other factors, such as gene length [3], tRNA abundance [43], and hydropathy of amino acid [32], have also been found to influence synonymous codon usage. For example, codon usage in the *Thermotoga maritima* genome was found to be the result of mutational bias, translation selection, the hydropathy of each gene, the anaerobic condition, and the usage of Cys [44]. In our study, COA and EST frequency were used to analyze the factors driving codon usage of *A. tamarense*. The factors involved in shaping codon usage of *A. tamarense* include at least the base composition at third codon positions and the expression level of each gene, as well as the hydropathy and the aromaticity of each gene.

The reasons why dinoflagellates contain this large amount of cellular DNA are still not known. Two opposite hypotheses, the “adaptive” versus the “junk” DNA hypotheses, respectively, have been proposed [35]. Our result showing the higher codon usage bias was found in highly expressed genes comparing with those of the protein CDSs in *A. tamarense* argues against the“junk” DNA hypothesis.

In summary, we have generated a collection of 4284 protein-coding genes, 74 ribosomal protein genes, and 40 plastid-related genes for dinoflagellate *A. tamarense* from 10685 ESTs. High mean Nc values of plastid-related genes and protein coding genes suggested their codon usage is mostly unbiassed. On the other hand, ribosomal protein genes exhibit strong codon usage bias. A codon usage-based strategy was applied to identify 11 highly expressed plastid-related genes and 17 translational optimal codons in *A. tamarense*. Our results suggest that mutational bias playing a major role in codon usage. However, gene expression level as well as the hydropathy and the aromaticity of genes are also likely to play role in this species. This might reflect the ecological success of dinoflagellates, which have large genome size and can grow very fast during red tide blooms, in the ecosystem.

## Figures and Tables

**Table 1:** The mean values and standard deviation of GC, GC1, GC2, GC3, and Nc for ribosomal protein genes, plastid-related genes, and other protein-coding genes in *Alexandrium tamarense* CCMP 1598 genes.

	N	GC (%)	GC1 (%)	GC2 (%)	GC3 (%)	NC
Ribosomal protein genes	74	60.41 ± 3.74	56.01 ± 6.66	41.93 ± 5.53	83.28 ± 7.37	39.99 ± 5.98
Plastid-related genes	40	60.78 ± 3.92	60.65 ± 5.52	43.64 ± 7.08	78.05 ± 7.77	42.17 ± 5.71
Other protein-coding genes	4170	61.98 ± 4.56	62.28 ± 6.73	45.70 ± 6.83	77.96 ± 8.15	43.64 ± 6.90

**Table 2:** Overall codon usage of *Alexandrium tamarense* CCMP 1598 genes. AA: amino acid; N: the number of codons. The preferentially used codons for each amino acid are displayed in bold.

AA	Codon	N	RSCU	AA	Codon	N	RSCU
Ala	GCU	10137	0.61	Ile	AUU	4320	0.60
	**GCC**	24014	1.45		**AUC**	15296	2.15
	GCA	12455	0.75		AUA	1764	0.25
	GCG	19668	1.19	Thr	ACU	3820	0.55
Gly	GGU	6589	0.52		ACC	9325	1.35
	**GGC**	28815	2.28		ACA	4071	0.59
	GGA	5724	0.45		**ACG**	10460	1.51
	GGG	9391	0.75	Asn	AAU	3336	0.44
Val	GUU	4886	0.47		**AAC**	11992	1.56
	GUC	15337	1.46	Lys	AAA	3143	0.20
	GUA	1760	0.17		**AAG**	28292	1.80
	**GUG**	19933	1.90	Tyr	UAU	2544	0.38
Phe	UUU	4949	0.46		**UAC**	10814	1.62
	**UUC**	16637	1.54	Cys	UGU	1939	0.32
Leu	UUA	607	0.06		**UGC**	10191	1.68
	UUG	9058	0.95	Asp	GAU	8318	0.50
	CUU	6427	0.67		**GAC**	24990	1.50
	CUC	17960	1.88	Glu	GAA	5918	0.27
	CUA	1143	0.12		**GAG**	37809	1.73
	**CUG**	22200	2.32	His	CAU	3460	0.49
Pro	CCU	5738	0.70		**CAC**	10620	1.51
	CCC	10406	1.26	Gln	CAA	3321	0.27
	CCA	6064	0.74		**CAG**	21528	1.73
	**CCG**	10677	1.30	Arg	CGU	3247	0.48
Ser	UCU	3832	0.59		**CGC**	13515	1.98
	UCC	9749	1.49		CGA	3447	0.51
	UCA	3167	0.49		CGG	9678	1.42
	UCG	7423	1.14		AGA	2479	0.36
	AGU	2706	0.42		AGG	8501	1.25
	**AGC**	12156	1.87				

**Table 3:** Comparison of codon usage frequencies between highly and lowly expressed sequences of *Alexandrium tamarense* CCMP 1598 genes. AA: amino acid; N: number of codons High: putatively highly expressed gene (10% of total genes with most negative value along axis 2 in Figure 3); Low: putatively low expressed gene (10% of total genes with most positive value along axis 2 in Figure 3). The codons displayed in bold are significantly more frequent between the highly expressed genes and the lowly expressed genes according to *χ* ^2^ test. Significant relationships are marked by: *P < .05; **P < .01; ***P < .001.

AA	Codon	High		Low		AA	Codon	High		Low	
		N	RSCU	N	RSCU			N	RSCU	N	RSCU
Ala	GCU	996	0.67	843	0.61	Ile	AUU***	583	0.78	286	0.51
	GCC	2167	1.45	1957	1.40		AUC	1596	2.14	1113	1.98
	GCA*	1282	0.86	1068	0.77		AUA	61	0.08	287	0.51
	GCG	1528	1.02	1702	1.22	Thr	ACU***	463	0.69	297	0.49
Gly	GGU***	778	0.65	470	0.44		ACC	935	1.40	775	1.29
	GGC	2795	2.34	2435	2.27		ACA**	468	0.70	341	0.57
	GGA	477	0.40	527	0.49		ACG	809	1.21	989	1.65
	GGG	734	0.61	861	0.80	Asn	AAU	328	0.43	329	0.50
Val	GUU***	536	0.56	366	0.41		AAC	1197	1.57	975	1.50
	GUC	1403	1.47	1251	1.42	Lys	AAA	309	0.18	346	0.24
	GUA	156	0.16	165	0.19		AAG	3066	1.82	2542	1.76
	GUG	1732	1.81	1752	1.98	Tyr	UAU	259	0.39	179	0.37
Phe	UUU*	517	0.50	366	0.43		UAC	1076	1.61	785	1.63
	UUC	1545	1.50	1335	1.57	Cys	UGU	195	0.34	138	0.28
Leu	UUA	37	0.05	59	0.07		UGC	941	1.66	842	1.72
	UUG***	885	1.12	696	0.86	Asp	GAU**	815	0.57	682	0.49
	CUU***	632	0.80	471	0.59		GAC	2058	1.43	2103	1.51
	CUC***	1607	2.03	1396	1.74	Glu	GAA	517	0.28	602	0.31
	CUA	58	0.07	135	0.17		GAG	3158	1.72	3322	1.69
	CUG	1530	1.93	2064	2.57	His	CAU	324	0.54	253	0.46
Pro	CCU	502	0.73	509	0.71		CAC	879	1.46	846	1.54
	CCC**	929	1.35	812	1.14	Gln	CAA	273	0.26	340	0.30
	CCA	556	0.80	566	0.79		CAG	1856	1.74	1921	1.70
	CCG	774	1.12	970	1.36	Arg	CGU***	609	1.36	64	0.12
Ser	UCU	339	0.62	305	0.56		CGC***	1335	2.97	470	0.91
	UCC***	1018	1.86	602	1.10		CGA	104	0.23	361	0.70
	UCA**	320	0.58	240	0.44		CGG	425	0.95	642	1.25
	UCG**	642	1.17	544	0.99		AGA	30	0.07	437	0.85
	AGU	224	0.41	261	0.48		AGG	190	0.42	1115	2.17
	AGC	745	1.36	1327	2.43						

## References

[1] Sharp PM, Li W-H (1986). An evolutionary perspective on synonymous codon usage in unicellular organisms. *Journal of Molecular Evolution*.

[2] Sharp PM, Stenico M, Peden JF, Lloyd AT (1993). Codon usage: mutational bias, translational selection, or both?. *Biochemical Society Transactions*.

[3] Duret L, Mouchiroud D (1999). Expression pattern and, surprisingly, gene length shape codon usage in *Caenorhabditis*, *Drosophila*, and *Arabidopsis*. *Proceedings of the National Academy of Sciences of the United States of America*.

[4] Karlin S, Mrázek J (1996). What drives codon choices in human genes?. *Journal of Molecular Biology*.

[5] Bulmer M (1991). The selection-mutation-drift theory of synonymous codon usage. *Genetics*.

[6] Rizzo PJ (2003). Those amazing dinoflagellate chromosomes. *Cell Research*.

[7] Uribe P, Fuentes D, Valdés J (2008). Preparation and analysis of an expressed sequence tag library from the toxic dinoflagellate *Alexandrium catenella*. *Marine Biotechnology*.

[8] Hégaret H, Wikfors GH, Soudant P (2007). Toxic dinoflagellates (*Alexandrium fundyense* and *A. catenella*) have minimal apparent effects on oyster hemocytes. *Marine Biology*.

[9] Lidie KB, Ryan JC, Barbier M, van Dolah FM (2005). Gene expression in Florida red tide dinoflagellate *Karenia brevis*: analysis of an expressed sequence tag library and development of DNA microarray. *Marine Biotechnology*.

[10] Bachvaroff TR, Place AR (2008). From stop to start: tandem gene arrangement, copy number and Trans-splicing sites in the dinoflagellate *Amphidinium carterae*. *PLoS ONE*.

[11] Bachvaroff TR, Concepcion GT, Rogers CR, Herman EM, Delwiche CF (2004). Dinoflagellate expressed sequence tag data indicate massive transfer of chloroplast genes to the nuclear genome. *Protist*.

[12] Hackett JD, Yoon HS, Soares MB (2004). Migration of the plastid genome to the nucleus in a peridinin dinoflagellate. *Current Biology*.

[13] Laatsch T, Zauner S, Stoebe-Maier B, Kowallik KV, Maier U-G (2004). Plastid-derived single gene minicircles of the dinoflagellate *Ceratium horridum* are localized in the nucleus. *Molecular Biology and Evolution*.

[14] Hackett JD, Anderson DM, Erdner DL, Bhattacharya D (2004). Dinoflagellates: a remarkable evolutionary experiment. *American Journal of Botany*.

[15] Erdner DL, Anderson DM (2006). Global transcriptional profiling of the toxic dinoflagellate *Alexandrium fundyense* using massively parallel signature sequencing. *BMC Genomics*.

[16] Rispe C, Legeai F, Gauthier J-P, Tagu D (2007). Strong heterogeneity in nucleotidic composition and codon bias in the pea aphid (*Acyrthosiphon pisum*) shown by EST-based coding genome reconstruction. *Journal of Molecular Evolution*.

[17] Kuo J, Chen M-C, Lin C-H, Fang L-S (2004). Comparative gene expression in the symbiotic and aposymbiotic *Aiptasia pulchella* by expressed sequence tag analysis. *Biochemical and Biophysical Research Communications*.

[18] Hackett JD, Scheetz TE, Yoon HS (2005). Insights into a dinoflagellate genome through expressed sequence tag analysis. *BMC Genomics*.

[19] Tanikawa N, Akimoto H, Ogoh K, Chun W, Ohmiya Y (2004). Expressed sequence tag analysis of the dinoflagellate *Lingulodinium polyedrum* during dark phase. *Photochemistry and Photobiology*.

[20] Cutter AD, Wasmuth JD, Blaxter ML (2006). The evolution of biased codon and amino acid usage in nematode genomes. *Molecular Biology and Evolution*.

[21] Sauvage C, Bierne N, Lapègue S, Boudry P (2007). Single Nucleotide polymorphisms and their relationship to codon usage bias in the Pacific oyster *Crassostrea gigas*. *Gene*.

[22] Ingvarsson PK (2008). Molecular evolution of synonymous codon usage in *Populus*. *BMC Evolutionary Biology*.

[23] Trivedi N, Bischof J, Davis S (2002). Parallel creation of non-redundant gene indices from partial mRNA transcripts. *Future Generation Computer Systems*.

[24] Gouzy J, Carrere S, Schiex T (2009). FrameDP: sensitive peptide detection on noisy matured sequences. *Bioinformatics*.

[25] Schiex T, Gouzy J, Moisan A, de Oliveira Y (2003). FrameD: a flexible program for quality check and gene prediction in prokaryotic genomes and noisy matured eukaryotic sequences. *Nucleic Acids Research*.

[26] Altschul SF, Madden TL, Schäffer AA (1997). Gapped BLAST and PSI-BLAST: a new generation of protein database search programs. *Nucleic Acids Research*.

[27] Wright F (1990). The ’effective number of codons’ used in a gene. *Gene*.

[28] Peden JF (1999). *Analysis of Codon Usage*.

[29] Greenacre MJ (1984). *Theory and Applications of Correspondence Analysis*.

[30] Musto H, Cruveiller S, D’Onofrio G, Romer H, Bernardi G (2001). Translational selection on codon usage in *Xenopus laevis*. *Molecular Biology and Evolution*.

[31] Bairoch A, Apweiler R, Wu CH (2005). The Universal Protein Resource (UniProt). *Nucleic Acids Research*.

[32] Liu Q (2006). Analysis of codon usage pattern in the radioresistant bacterium *Deinococcus radiodurans*. *BioSystems*.

[33] Akashi H (1996). Molecular evolution between *Drosophila melanogaster* and *D. simulans*: reduced codon bias, faster rates of amino acid substitution, and larger proteins in *D. melanogaster*. *Genetics*.

[34] Liu Q, Xue Q (2005). Comparative studies on codon usage pattern of chloroplasts and their host nuclear genes in four plant species. *Journal of Genetics*.

[35] Peixoto L, Zavala A, Romero H, Musto H (2003). The strength of translational selection for codon usage varies in the three replicons of *Sinorhizobium meliloti*. *Gene*.

[36] Grocock RJ, Sharp PM (2002). Synonymous codon usage in *Pseudomonas aeruginosa* PA01. *Gene*.

[37] Romero H, Zavala A, Musto H, Bernardi G (2003). The influence of translational selection on codon usage in fishes from the family Cyprinidae. *Gene*.

[38] Stenico M, Lloyd AT, Sharp PM (1994). Codon usage in *Caenorhabditis elegans*: delineation of translational selection and mutational biases. *Nucleic Acids Research*.

[39] Hou Y, Lin S (2009). Distinct gene number-genome size relationships for eukaryotes and non-eukaryotes: gene content estimation for dinoflagellate genomes. *PLoS ONE*.

[40] McEwan M, Humayun R, Slamovits CH, Keeling PJ (2008). Nuclear genome sequence survey of the dinoflagellate *Heterocapsa triquetra*. *Journal of Eukaryotic Microbiology*.

[41] Koumandou VL, Nisbet RER, Barbrook AC, Howe CJ (2004). Dinoflagellate chloroplasts—where have all the genes gone?. *Trends in Genetics*.

[42] Howe CJ, Barbrook AC, Lockhart PJ (2000). Organelle genes—do they jump or are they pushed?. *Trends in Genetics*.

[43] Percudani R, Pavesi A, Ottonello S (1997). Transfer RNA gene redundancy and translational selection in *Saccharomyces cerevisiae*. *Journal of Molecular Biology*.

[44] Zavala A, Naya H, Romero H, Musto H (2002). Trends in codon and amino acid usage in *Thermotoga maritima*. *Journal of Molecular Evolution*.

